# Design of Dosage Forms with Improved Biopharmaceutical Properties

**DOI:** 10.3390/pharmaceutics16010069

**Published:** 2024-01-02

**Authors:** Nataliya Kochkina, Irina Terekhova

**Affiliations:** G.A. Krestov Institute of Solution Chemistry of RAS, 153045 Ivanovo, Russia; nek@isc-rus.ru

The development of a pharmaceutical product consists of giving a drug an optimal dosage form (a certain state of aggregation, consistency, structural, mechanical, physicochemical, and functional properties), which ensure stability, the possibility of accurate dosage, the required pharmacological effect, and ease of administration with minimal side effects [1,2,3]. Modern pharmacology pays special attention to improving the biopharmaceutical properties of dosage forms through innovative methods. This approach represents a growing trend aimed at expanding therapeutic options for ensuring optimal patient outcomes.

This Special Issue “Design of Dosage Forms with Improved Biopharmaceutical Properties” features valuable contributions from researchers in this area of pharmaceutical science. Nine original papers comprise the issue, covering the development of potential theranostic agents (contributions 1 and 2), polysaccharide nanoparticles (contributions 3 and 4), beads loaded with drugs (contribution 5), pharmaceutical gels (contribution 6), metal–organic frameworks (contribution 7), micellar systems (contribution 8), and drug nanoformulations (contribution 9) with advanced biopharmaceutical characteristics.

Theranostics is an emerging discipline within personalized medicine, which encompasses the utilization of agents with dual action in both diagnosing and inhibiting the proliferation of cancer cells. This Special Issue includes two papers focused on the development of such systems. In a study conducted by Akhmetov et al. (contribution 1), a novel approach towards regioselective functionalization of thiacalix[4]arene with a fluorescent label was suggested to synthesize mimics of antiangiogenic agents. They uncovered that these molecular structures can penetrate both viable and non-viable A549 and HuTu-80 cancer cells, implying potential utility for treating tumor neoplasms and tracking the delivery and biodistribution of nanomedicines. In another study, multimodal contrast agents were produced using carbon-coated manganese ferrite nanoparticles, which were synthesized via a hydrothermal method and then encapsulated in oxidized sodium alginate (contribution 2). The findings indicated that these agents exhibited higher biocompatibility and lower toxicity compared to the traditional contrast agents. Moreover, they were found to be capable of sustained release of doxorubicin hydrochloride, a drug employed in cancer therapy. The authors concluded the potentiality of these systems as theranostic agents for multimodal diagnosis and cancer treatment.

Sodium alginate, a FDA-approved polysaccharide [4], is frequently used in the formulation of novel dosage forms due to its excellent biodegradability, biocompatibility, and water solubility features. As shown by Bhosale et al. (contribution 3), when sodium alginate is applied to mucoadhesive chitosan nanoparticles, there is an increase in voriconazole loading capacity and in the ability of these nanoparticles to penetrate mucosa. These systems are potential candidates for use as nanocarriers in treating fungal keratitis as they exhibit improved release kinetics and corneal permeation in comparison to the pristine chitosan nanoparticles. In the research of Uthumansha et al. (contribution 5), a bead formulation was developed using the emulsion gelation method to encapsulate telmisartan, an antihypertensive drug, within alginate. These beads demonstrated high entrapment efficiency and long-term antihypertensive effects, as was confirmed with in vivo experiments.

Chitosan, also a polysaccharide, is commonly utilized to formulate nanoparticles for drug delivery systems [5]. Sorasitthiyanukarn et al. (contribution 4) presented a study on the development of hexahydrocurcumin-encapsulated chitosan nanoparticles, as an alternative therapeutic agent for breast cancer treatment. Notably, these nanoparticles were shown to have improved bioaccessibility, bioavailability, and exhibited increased antioxidant and anti-inflammatory activities compared to the free hexahydrocurcumin. They also showed that the designed nanoparticles display superior protein anti-denaturation activity and high cytotoxicity towards MDA-MB-231 breast cancer cells.

The use of drugs for cancer treatment is often accompanied by serious side effects. To minimize the adverse effects associated with the systemic use of cytostatic methotrexate [6], the authors of contribution 6 suggested the application of a topical hydrogel based on kappa-carrageenan. β-Cyclodextrin was added in this gel as an additive allowing the achievement of a 10-fold increase in the drug solubility. The study demonstrated that the amount of methotrexate released from the gels can be controlled by adjusting the concentration of β-cyclodextrin. The rheological properties of the hydrogel were also investigated, and it was found that the gel with β-cyclodextrin content is easier to apply on skin or tissues compared to the gel without cyclodextrin.

Increasing solubility and bioavailability of a drug is one of the frequently solved problems in the development of pharmaceuticals with improved biopharmaceutical properties. This problem is addressed by the authors of contribution 7. Metal–organic frameworks (MOFs) are porous materials composed of metal nodes or clusters and organic ligands linked by coordination bonds [7]. The use of cyclodextrins as natural and safe linkers makes MOFs biocompatible and promising materials for biomedical applications. The authors of contribution 7 used a metal–organic framework based on γ-cyclodextrin (γCD-MOF) as a carrier for non-steroidal anti-inflammatory drugs (NSAIDs)**.** The main regularities of the entrapment of NSAIDs in γCD-MOF were revealed. After analyzing the impact of the lipophilicity and the polar surface area of the NSAIDs molecules, as well as their ability to ionize, the authors found that the drug loading is mainly dependent on log*P*. As demonstrated, NSAIDs loaded in γCD-MOF have improved dissolution profiles.

The strategy for increasing the solubility and therapeutic effect of the hepatoprotective drug baicalin by integrating it into the micelles of diammonium glycyrrhizinate is proposed in contribution 8. Diammonium glycyrrhizinate is a biosurfactant displaying anti-inflammatory and liver protection effects [8]. The formation of such micelles resulted in an increase in the cumulative percentage release of baicalin in the gastrointestinal tract. In vivo experiments demonstrated the synergistic hepatoprotective effect of baicalin integrated into diammonium glycyrrhizinate micelles compared to the direct combined use of these two drugs.

In contribution 9, the dry co-milling technology was employed to enhance the solubility of the drug celecoxib. In vitro and in vivo studies showed that the nanoformulations produced by co-milling celecoxib with povidone, mannitol, and sodium lauryl sulfate exhibited a better dissolution rate and oral pharmacokinetic profile compared to commercial Celebrex.

In conclusion, recent studies have shown that the design of the dosage forms with improved properties can be the basis for the development of innovative medicinal products and increase patients’ access to modern therapies.

The editors of this Special Issue would like to express their gratitude to all the authors and reviewers who have contributed their time and expertise and helped to make this collection possible.
